# Editorial Note: Does an improved HDI trigger tourism outflows for China? New evidence from the ARDL cointegration approach

**DOI:** 10.1371/journal.pone.0351364

**Published:** 2026-06-11

**Authors:** The PLOS One Editors

The *PLOS One* Editors issue this Editorial Note to inform readers of the following issues that were noted after this article’s [1] publication:

- The work cited in this article [1] as Reference 24 was retracted after the article’s publication date. PLOS considers that this reference is not crucial in supporting the research or conclusions reported in [1].
- PLOS has concerns about the article’s authorship.
